# Advancing Cancer Drug Delivery with Nanoparticles: Challenges and Prospects in Mathematical Modeling for In Vivo and In Vitro Systems

**DOI:** 10.3390/cancers17020198

**Published:** 2025-01-09

**Authors:** Tozivepi Aaron Munyayi, Anine Crous

**Affiliations:** Laser Research Centre, Faculty of Health Sciences, University of Johannesburg, P.O. Box 17011, Doornfontein 2028, South Africa

**Keywords:** cancer, nanoparticles, in vitro, in vivo, therapy, drug resistance, preclinical, overestimation

## Abstract

Mathematical models are crucial for predicting the behavior of drug conjugate nanoparticles and optimizing drug delivery systems in cancer therapy. These models simulate interactions among nanoparticle properties, tumor characteristics, and physiological conditions, including drug resistance and targeting specificity. However, they often rely on assumptions that may not accurately reflect in vivo conditions. In vitro studies, while useful, may not fully capture the complexities of the in vivo environment, leading to an overestimation of nanoparticle-based therapy effectiveness. Advancements in mathematical modeling, supported by preclinical data and artificial intelligence, are vital for refining nanoparticle-based therapies and improving their translation into effective clinical treatments.

## Simple Summary

Cancer treatment can be improved by using tiny particles, called nanoparticles, to deliver drugs directly to cancer cells. To understand how these drug-loaded nanoparticles work in the body, researchers create mathematical models that predict their behavior. These models help us understand how different factors—like the size and makeup of nanoparticles, the characteristics of tumors, and the body’s own responses—affect the treatment’s success. However, current models sometimes make assumptions that may not match what happens in real-life scenarios, causing us to overestimate the treatment’s effectiveness. This research aims to refine these mathematical models by incorporating more realistic data and advanced artificial intelligence techniques. These improvements may lead to more accurate predictions and ultimately make nanoparticle-based cancer treatments more successful in clinical settings, benefiting patients and advancing the field of cancer therapy.

## 1. Introduction

Drug delivery is crucial in cancer therapy, influencing both the efficacy and safety of treatments [1,2,3,4]. Traditional methods, such as systemic chemotherapy, distribute therapeutic agents throughout the body, targeting both cancerous and healthy cells, often resulting in significant side effects due to the lack of specificity [5,6,7]. Advanced delivery systems, such as nanoparticles (NPs) and targeted therapies, aim to improve this by offering more precise and controlled delivery, reducing systemic toxicity, and enhancing treatment effectiveness [2,7,8].

Despite their potential, NP-mediated cancer therapies have only seen moderate clinical success, primarily due to low tumor delivery efficiency [9,10,11]. Although NPs can exploit the enhanced permeability and retention (EPR) effect and actively target cancer cells via surface functionalization, only about 0.7% of the injected dose accumulates in tumors [12,13]. Enhanced permeability and retention (EPR) refers to the tendency of nanoparticles or drugs to accumulate more in tumor tissues than in normal tissues due to the leaky blood vessels and poor lymphatic drainage in tumors [14]. EPR efficiency measures the effectiveness of this phenomenon in delivering therapeutic agents to tumor sites, with higher efficiency indicating better accumulation and retention of the drug in the tumor [15]. Inefficiency is largely due to rapid clearance by the mononuclear phagocytic system (MPS) and kidneys, which is influenced by NP size, charge, and the physiological characteristics of these organs [5,16,17]. Understanding and manipulating these factors are crucial to improving NP delivery to tumors and enhancing therapy success.

To better navigate these challenges, mathematical and computational models have emerged as powerful tools for predicting NP behavior, guiding experimental design, and refining therapeutic strategies. These models integrate principles from physics, chemistry, and biology to simulate NP transport, distribution, and interactions at various levels—ranging from molecular events at the cell membrane to systemic factors like blood flow and organ clearance. By incorporating experimental data, such as NP physicochemical characteristics, preclinical animal studies, and patient-derived tumor parameters, mathematical models can provide insights into how altering NP properties or administering specific dosing regimens might enhance tumor accumulation and improve drug delivery efficiency. Such models also help identify critical variables that influence treatment outcomes, enabling researchers to focus on optimizing key parameters like NP size, shape, and surface functionalization. Furthermore, advancements in computational techniques, including machine learning and multiscale modeling, allow for more accurate, patient-specific predictions, potentially accelerating the translation of NP-based therapies from the laboratory to clinical practice.

Conversely, the inefficiencies of NP-mediated therapies, such as poor targeting accuracy, inadequate drug release, and limited tissue penetration, stem from the complex interactions between NPs and biological systems, which current mathematical models fail to fully capture [2,18]. These models often oversimplify biological environments, neglecting factors like tissue heterogeneity, immune responses, and patient variability, which limits their predictive power. As a result, NP designs cannot be optimized effectively, leading to suboptimal therapeutic outcomes and hindering clinical translation [19,20]. To improve NP-based therapies, more sophisticated, multiscale, and personalized mathematical models are needed to better simulate NP behavior and address these inefficiencies.

## 2. Challenges in In Vivo and In Vitro Nano-Based Cancer Drug Delivery

Challenges such as drug resistance, targeting specificity, and the tumor microenvironment significantly hinder the effectiveness of nano-based cancer therapies [1,2,4,21]. Drug resistance occurs when cancer cells adapt to evade the effects of treatment, often through genetic mutations or by activating alternative survival pathways, making it difficult to achieve long-term remission [21,22,23,24]. Targeting specificity is another critical challenge, as ensuring that drug delivery systems accurately distinguish between cancerous and healthy cells is essential to minimize side effects and enhance therapeutic efficacy [25,26,27,28]. The tumor microenvironment, characterized by abnormal vasculature, high interstitial pressure, and a dense extracellular matrix, further complicates treatment by impeding drug penetration and distribution within the tumor [29,30,31]. Addressing these challenges requires innovative strategies that can overcome biological barriers, enhance targeting precision, and counteract resistance mechanisms to improve cancer treatment outcomes. Furthermore, developing dynamic models that consider time-dependent changes in tumor microenvironments and physiological conditions can offer more realistic simulations of nanoparticle behavior throughout treatment [32,33]. These models would better reflect the complexities of how tumors evolve and how the body responds over time, leading to more accurate predictions and optimized treatment strategies [34,35]. By incorporating these dynamic factors, the models can enhance the precision of drug delivery systems and improve their effectiveness in cancer therapy.

**Figure 1:**
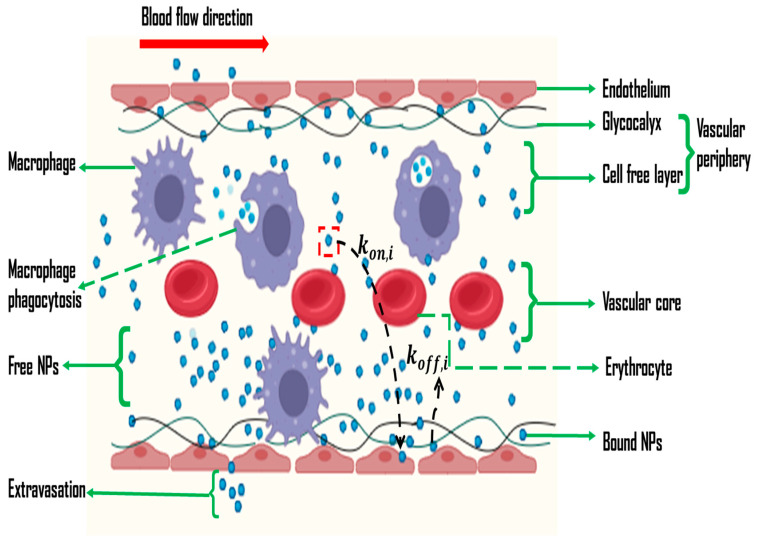
Schematic diagram showing the NP adsorption and dislodgement during the delivery process.

The human body is far more complex than controlled lab conditions, where in vitro studies are typically conducted in simplified environments that do not fully replicate the intricate interactions within a living organism, such as the immune system, blood flow dynamics, and the presence of various cell types and tissues [36,37]. In vivo, NPs must navigate various biological barriers, including the MPS, blood–brain barrier, and the complex tumor microenvironment, which can significantly reduce the efficiency of NP delivery to the target site (Figure 1) [38]. Additionally, NPs in vivo are often rapidly cleared from the bloodstream by organs like the liver and spleen, reducing their availability to target tumors [12]. The distribution of NPs throughout the body (biodistribution) can also be unpredictable, leading to off-target effects and reduced efficacy at the tumor site [12,38]. Furthermore, tumors in vivo have highly heterogeneous microenvironments that vary greatly from patient to patient and even within different regions of the same tumor, affecting how NPs interact with the tumor and leading to inconsistent drug delivery and therapeutic outcomes [12,31,38]. Finally, the immune system in vivo can recognize and eliminate NPs before they reach the tumor, a factor that in vitro studies typically do not account for, leading to an overestimation of NP efficacy [36]. Several assumptions in drug delivery systems have led to the overestimation of efficacy in real-life applications. For example, the assumption that smaller nanoparticles penetrate tumors more effectively has been challenged by tumor heterogeneity and blood vessel permeability, which limit nanoparticle penetration [39,40]. Similarly, the EPR effect, often relied upon for targeted drug delivery, fails in tumors with well-organized blood vessels, reducing nanoparticle accumulation. Additionally, the assumptions of stable, controlled drug release can be disrupted by factors like pH changes and enzymatic activity, leading to premature drug release. Lastly, surface modifications, such as targeting ligands, are not always effective due to receptor expression variability and immune system interactions [41,42]. These cases highlight the need for more realistic models to address the complexities of drug delivery systems. Incorporating comprehensive preclinical data into mathematical models can significantly improve their accuracy and predictive power [43,44]. This data, derived from various animal models, help capture the diversity of tumor types and treatment responses, providing a more robust understanding of how nanoparticles behave in different scenarios. By including this broad range of preclinical insights, models can better simulate real-world conditions, leading to more effective predictions and optimized drug delivery strategies for cancer therapy [45]. Furthermore, regulatory hurdles, scalability factors, and patient-specific factors impact the efficacy of NP-based drug delivery. Strict regulatory standards can delay adoption, while scaling production for clinical use may affect consistency and cost-effectiveness [46,47]. Additionally, individual patient factors (genetics, health conditions, and immune responses) can influence how NPS are absorbed and metabolized, further complicating treatment effectiveness [48].

In addition to the complexities encountered in vivo, the in vitro evaluation of nanoparticle-based therapies also presents inherent challenges. These include the use of simplified, two-dimensional cell culture systems that fail to mimic the three-dimensional architecture, extracellular matrix composition, and dynamic fluid flows of tumor tissues, potentially leading to inaccurate assessments of nanoparticle penetration and uptake [49]. Moreover, standard in vitro assays often lack critical components such as immune cells, stromal cells, and vascular networks, thereby overlooking key biological interactions and responses that influence nanoparticle behavior in patients [50]. These limitations can result in the overestimation of therapeutic efficacy, as in vitro conditions do not capture the immune clearance, metabolic variability, and heterogeneous microenvironments that NPs encounter in vivo. Consequently, more complex in vitro models, including three-dimensional spheroid or organoid cultures and microfluidic “tumor-on-a-chip” devices, are being explored to improve the predictive power of preclinical studies and better inform the design of effective nanoparticle-based cancer therapies [51].

Figure 1 illustrates a model applied in both in vivo and in vitro cancer drug delivery systems, focusing on the vascular wall and the influence of NP properties on drug delivery dynamics [16,38]. It demonstrates how the size, shape, and surface chemistry of NPs significantly affect the rates of NP adsorption ($k_{on,i}$) and dislodgement ($k_{off,i}$) during the delivery process [52,53]. This visualization underscores the importance of NP design in optimizing drug delivery efficacy and overcoming the challenges associated with both in vivo and in vitro systems [54,55]. Smaller NPs typically have higher diffusion rates, which can increase their ability to approach and bind to the endothelial wall, potentially enhancing $k_{on,i}$ [53]. Conversely, larger NPs might face greater resistance in diffusing through the glycocalyx and therefore could exhibit reduced $k_{on,i}$ and $k_{off,i}$ [56].

The shape of NPs also affects their interaction with the endothelial surface, with shapes that align better with the wall geometry possibly enhancing the binding and affecting the dislodgement rate [55]. Surface chemistry, including functional groups and charge, influences the binding affinity and stability of NPs on the endothelial wall, altering both $k_{on,i}$ and $k_{off,i}$ [53]. Surface modifications can enhance specific interactions or promote repulsion, thus impacting how readily NPs adhere to or dislodge from the endothelial surface [57]. These factors collectively influence the efficiency and effectiveness of NP-mediated drug delivery in vitro systems.

Mathematical models are key to predicting the behavior of drug conjugate nanoparticles and optimizing drug delivery systems in cancer therapy. While challenges such as the assumptions and limitations of in vitro studies exist, advancements in mathematical modeling, particularly when combined with preclinical data and AI, show great potential for improving nanoparticle-based therapies [44]. AI has been integrated into both in vitro and in vivo nanoparticle drug delivery models, improving drug targeting and delivery accuracy [58]. In vitro, AI analyzes experimental data to optimize nanoparticle design for better cellular uptake and drug release. Machine learning, including deep learning, helps predict cell responses and nanoparticle interactions, enabling personalized delivery strategies [59]. In vivo, AI simulates complex biological systems, accounting for tumor heterogeneity and immune responses to predict nanoparticle behavior [60]. Overall, AI bridges the gap between theoretical models and clinical applications, enhancing the efficacy of nanoparticle-based therapies in cancer treatment. These models can enhance prediction accuracy and aid in translating research into clinical treatments, advancing cancer therapy and patient outcomes. To address these challenges, a comprehensive understanding of NP behavior within systemic pharmacokinetics and the tumor microenvironment is essential [12,38,52]. Preclinical studies have highlighted various physicochemical and physiological factors, but a holistic approach using mathematical models is needed to simulate the complex interactions between NP properties, tumor characteristics, and physiological conditions [61,62]. Existing models have provided valuable insights but are often limited in scope or lack tumor-specific compartments [62,63]. Advancing mathematical models for NP-based drug delivery can improve the design, predict behavior, and enhance therapeutic efficacy, leading to more effective and targeted cancer treatments [61].

In conclusion, this review delves into the complexities and potential advancements in the mathematical modeling of nanomedicine, providing a comprehensive examination of how the biophysicochemical properties of nanoparticles influence drug delivery effectiveness. By analyzing the interplay between these properties and various biological conditions, both in vivo and in vitro, this review aims to highlight the current challenges and prospects in refining nanoparticle-based therapies. Through a detailed exploration of model limitations and advancements, including the integration of preclinical data and artificial intelligence, this review seeks to offer valuable insights into optimizing drug delivery systems and enhancing their clinical translation. Furthermore, there is a significant need for multi-scale modeling approaches that link molecular interactions to cellular and tissue-level responses. These models may offer a more comprehensive understanding of how drug conjugate nanoparticles behave in complex biological systems, improving the design and effectiveness of drug delivery systems in cancer therapy by capturing the full spectrum of nanoparticle behavior.

## 3. Nanoparticle-Based Cancer Drug Delivery Models

**Figure 2:**
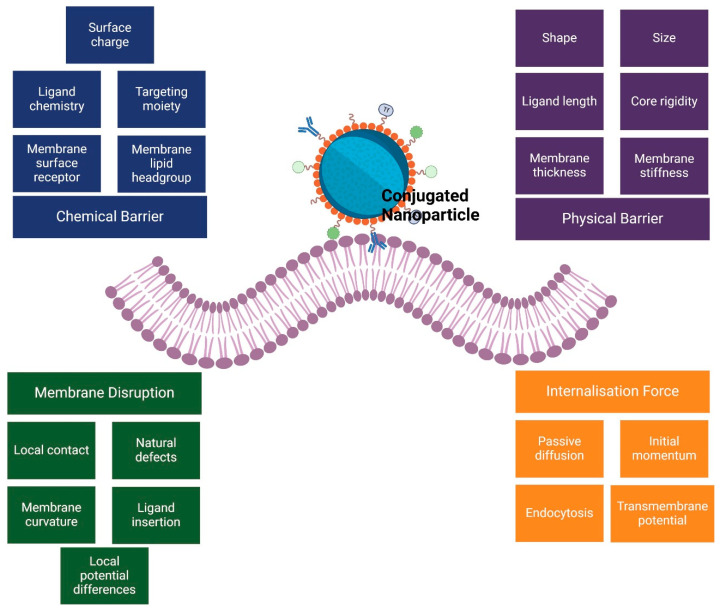
The mechanisms of nanoparticle interaction with cell membranes: a framework of chemical and physical barriers.

This section explores the common modeling approaches used to describe and predict the behavior of nanoparticle-based drug delivery systems in cancer therapy. Figure 2 illustrates the complex interaction between nanoparticles and cell membranes by highlighting the dynamic interplay of four key factors. Physical and chemical barriers at the cell membrane level, such as lipid and protein composition [16], membrane charge, and local molecular environment [53], can impede or facilitate nanoparticle penetration. Internalization forces, including endocytosis [64,65] and membrane disruption [65,66] also influence how effectively nanoparticles move from the bloodstream into target cells and tissues. When considering the movement of nanoparticles within the vasculature, factors such as nanoparticle size, shape, surface charge, and ligand chemistry, as well as fluid dynamics and red blood cell (RBC)-induced shear forces, collectively govern their radial migration from the vessel core toward the vessel walls. For instance, nanoparticle margination—the tendency of nanoparticles to drift toward the periphery of the blood vessel—depends on their physicochemical properties [54,65,66] and their interactions with flowing blood components [55,61,67]. This margination process is further influenced by the endothelial glycocalyx [55,61], which can either facilitate or hinder nanoparticle adhesion depending on nanoparticle surface characteristics and the local vascular environment. Ultimately, these biophysicochemical factors and their interplay determine how nanoparticles are distributed, deposited, and ultimately taken up by cells, thus impacting their therapeutic efficacy [54,65,66].

Nanoparticles within the microvascular space often exhibit a non-uniform radial distribution, showing a propensity to move from the vascular core to the periphery [55]. This distribution is influenced by Brownian motion, shear-induced diffusion due to erythrocytes, and sedimentation [61,67]. Once at the periphery, interactions with the endothelial glycocalyx and vessel wall determine whether the nanoparticles remain free-flowing or adhere to the surface. This adhesion process is a critical step that directs nanoparticles closer to target tissues. The overall deposition of nanoparticles on the microvascular wall is thus governed by complex interactions among nanoparticle characteristics, hemorheological parameters, and hemodynamic conditions which collectively influence nanoparticle migration, distribution, and kinetics at the organ level [55,67,68].

Assuming the nanoparticle motion from the core toward the vessel wall is governed by gravitational and buoyant forces (Figure 1), the terminal sedimentation velocity $v_{t}$ of a nanoparticle with radius r can be derived using Stoke’s law. This simplified calculation focuses solely on sedimentation by disregarding longitudinal blood flow and RBC presence, providing a baseline understanding of nanoparticle behavior before incorporating additional complexities of the blood microenvironment [55,61]. Stoke’s law (Equation (1)) provides the terminal velocity of a spherical NP settling in a fluid due to gravity:

(1) $$v_{t}=\frac{{2r}^{2}\left(\rho_{NP}-\rho_{f}\right)g}{9\eta_{f}}$$

where: $v_{t}$ is the terminal sedimentation velocity, r is the radius of the nanoparticle, $\rho_{p}$ is the density of the nanoparticle, $\rho_{f}$ is the density of the fluid (blood plasma), g is the gravitational acceleration constant, and $\eta_{f}$ is the dynamic viscosity of the fluid. The equation calculates the sedimentation rate of a NP under gravitational forces, considering the NP’s size and the fluid properties, and assumes the NP operates in a low Reynolds number $\left(R=\frac{{l.u.\rho }_{f}}{\eta_{f}}<1\right)$ regime, where viscous forces dominate over inertial forces [69].

However, in the presence of blood flow and erythrocytes, the sedimentation velocity of NPs is adjusted by dividing the original equation (Equation (1)) by the characteristic length of sedimentation (the radius of the microvessel, *R*) and the Peclet number $\left(Pe=\left(\frac{Dl}{uR^{2}}\right)\right)$ [70]. The Peclet number is calculated using the average blood flow velocity and the effective diffusivity of NPs, which includes contributions from both Brownian motion ($D_{B}$) derived from the Stokes-Einstein equation and shear-induced diffusion ($D_{s}$) based on an analytical expression by Xu et al. [71].

(2) $$D=\frac{kTC_{c}}{6\pi r\eta_{f}}+0.3\beta^{2}\gamma H^{2}$$

where *k* is the Boltzmann’s constant (1.38 × 10^−23^ J K^−1^), *T* is the absolute temperature in K, $\eta_{f}$ is the dynamic viscosity of fluid, *r* is the nanoparticle radius, *C_c_* is a slip correction factor, *β* is the radius of the erythrocytes, *γ̇* is the shear rate, and *H* is the hematocrit: the volume fraction of red blood cells in the blood.

Subsequently, the final adjustment results in the $k_{on,i}$ parameter (${time}^{-1}$), which characterizes the rate of deposition of the NPs on the microvascular wall, as depicted by Equation (3).

(3) $$k_{on,i}=\frac{{2r}^{2}\left(\rho_{NP}-\rho_{f}\right)g}{9\eta_{f}}\times \frac{Dl}{uR^{3}}$$

NPs marginated towards the microvascular wall are either endocytosed by macrophages or remain non-specifically bound to the endothelial wall [61]. However, NPs can re-enter the vessel lumen and continue their transport if they diffuse back through the endothelial glycocalyx’s characteristic diffusion length (*l_g_*), with the rate of dislodging $\left(k_{off,i}\right)$ determined by their ability to traverse this glycocalyx layer, as highlighted by Equation (4) [55,72].

(4) $$k_{off,i}=\frac{D_{B}}{l_{g}^{2}}$$

Although NP dislodgement from the endothelial wall can be influenced by specific interactions between NP surface ligands and endothelial cell receptors (Figure 2), many studies simplify this by assuming that the relative tendency of NPs to approach and diffuse away from the wall is governed by $k_{on,i}$ and $k_{off,i}$, respectively [12,53,55,67].

When nanoparticles approach a cell membrane, they may adsorb, penetrate, or be internalized via endocytosis, depending on their surface characteristics and the membrane’s composition [54,65,66]. Basic models developed to study these interactions often focus on electrostatic forces, van der Waals forces, and hydrophobic interactions, which govern the adsorption and adhesion of nanoparticles to the cell membrane [62,73,74,75]. Additionally, molecular dynamics simulations and continuum models have been employed to predict the behavior of nanoparticles at the nanoscale, providing insights into how factors like nanoparticle rigidity and ligand–receptor binding can influence cellular uptake [75,76,77]. These models help in understanding the initial stages of nanoparticle–membrane interaction and are essential for optimizing drug delivery efficiency and minimizing off-target effects [78,79].

**Figure 3:**
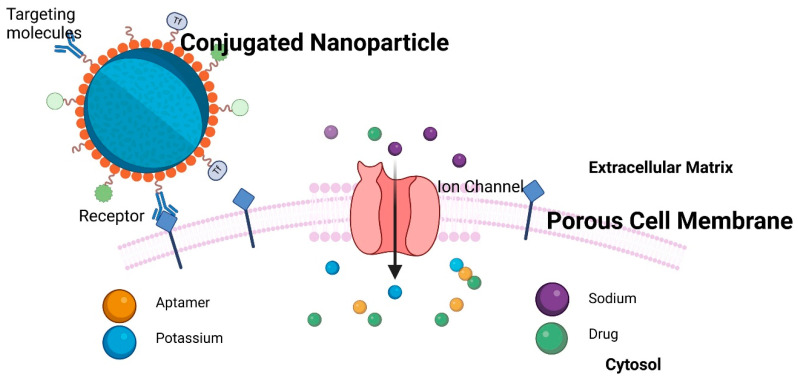
Conjugated nanoparticle–membrane interactions.

Figure 3 depicts the interactions of nanoparticles with porous membranes, which are a focal point in various theoretical studies including drug delivery mathematical models [16,55]. These interactions are crucial for understanding how NPs navigate and permeate biological barriers, influencing their effectiveness in drug delivery systems.

Various models have been developed to simulate how conjugated nanoparticles interact with cellular membranes, especially focusing on their behavior as they encounter and interact with membrane pores [62,73,80,81]. These models help in understanding and predicting the efficiency and mechanisms of nanoparticle delivery into cells. Goldberg et al. conducted a comprehensive review of the models used to describe nanoparticle transport in saturated porous media, evaluating their ability to predict flow [82]. The models were categorized according to key transport phenomena, including flow equations, deposition terms, remobilization, and blocking effects [82]. Notably, the study suggested that increased model complexity does not necessarily correlate with improved predictive accuracy for nanoparticle behavior.

Ju and Fan developed a mathematical model for nanoparticle transport in porous media with several key assumptions: (1) the flow is one-dimensional and isothermal with incompressible fluids; (2) the porous medium is heterogeneous; (3) both drug and nanoparticle colloid flow follow Darcy’s law, with gravity effects ignored; (4) nanoparticles are divided into discrete intervals; and (5) fluid viscosity and density are constant, and the medium behaves as a Newtonian fluid [83]. The model employs Darcy’s law to describe fluid flow in porous media, represented by Equation (5):

(5) $$\frac{\partial }{\partial t}\left(\emptyset S_{l}\right)-\frac{\partial }{\partial x}\left(\frac{K_{l}{\partial P}_{l}}{\mu_{l}\partial x}\right)=0;l=0,w,$$

where *x* is the distance of the particle in the reservoir, *t* is time, *ϕ* is the porosity of the reservoir medium, *K*_*l*_ is the effective permeability, and *S*_*l*_, *μ*_*l*_, and *P*_*l*_ are the saturation, viscosity, and porosity of phase *l* (media), respectively. Ju and colleagues developed models to describe the changes in porosity and the permeability of porous media following the injection of cancer drug-conjugated nanoparticles [84]. These changes are mathematically represented by Equations (6) and (7), which quantify how nanoparticle injection alters the structural and flow properties of the porous media, respectively:

(6) $$\emptyset =\left(\emptyset_{0}-\sum ∆\emptyset \right)$$

The permeability of the porous medium, after the injection of cancer drug-conjugated nanoparticles, can be calculated using Equation (7):

(7) $$K=K_{0}{\left[\left(1-f\right)k_{f}+\frac{f\emptyset }{\emptyset_{0}}\right]}^{n}$$

The model, solved using the implicit pressure explicit saturation (IMPES) method, accurately predicts nanoparticle movement in porous media [85]. As one of the pioneering models for evaluating nanoparticle transport, it has been widely utilized and referenced in subsequent research advancements [86,87,88]. Most NP-based drug delivery mathematical models rely on the conceptual framework depicted in Figure 1 and Equations (1)–(7), with minor modifications to address specific target niches. However, these equations often necessitate cumbersome and continuous optimization to account for the complex differences between in vivo and in vitro scenarios, reflecting the challenges in achieving accurate and universal modeling for diverse nanoparticle physicochemical properties and biological environments. Furthermore, while nanoparticle-based cancer drug delivery models complement each other and provide valuable insights, their predictive power for in vivo efficacy remains limited. This gap highlights the need for continued refinement and the integration of experimental data to enhance model accuracy and better translate laboratory successes into effective clinical outcomes.

## 4. Future Prospects

While mathematical models are excellent at predicting nanoparticle-based cancer drug delivery, they are often built on assumptions that may not be feasible in vivo. These models typically simplify the complexities of the human body, such as the variability in biological environments, the dynamic interactions between nanoparticles and biological systems, and the impact of the immune response [89,90]. A critical challenge in developing these models is their validation against experimental data. Ensuring that models accurately reflect biological realities is essential for their utility in clinical applications. As a result, the predictions made by these models may not fully capture the challenges encountered during actual in vivo drug delivery.

Moreover, mathematical modeling plays a crucial role in the design and development of next-generation drug delivery systems by allowing researchers to explore a wide range of scenarios and predict outcomes before conducting expensive and time-consuming experiments [91,92]. However, future improvements lie in the integration of diverse data sources and advanced computational techniques to increase the fidelity of these models. By incorporating high-resolution imaging data, multi-omic datasets (genomic, transcriptomic, proteomic, and metabolomic), patient-specific tumor characteristics, and real-time clinical feedback, models can begin to approximate the complexities of the tumor microenvironment and systemic circulation more accurately. Such data-rich models can better capture dynamic processes, including tumor evolution, metastasis, and the interplay between cancer cells, immune cells, and the vasculature. Additionally, advances in artificial intelligence (AI) and machine learning are poised to further enhance the predictive power of mathematical models in nanoparticle-based delivery [93,94,95]. AI-driven approaches can help identify patterns and relationships that are difficult to discern through traditional modeling methods, leading to improved parameter estimation, model calibration, and uncertainty quantification. This can facilitate the rapid adaptation of models as new data become available, making it possible to tailor therapies in a patient-specific manner and improve treatment outcomes. The development of hybrid models that combine mechanistic understanding with data-driven insights holds particular promise in refining dosing strategies, selecting the most effective nanoparticle formulations, and predicting therapeutic responses more accurately [96].

On the engineering side, emerging fabrication techniques, such as microfluidic templating and bioprinting, can produce nanoparticles with more precise control over size, shape, and surface chemistry. These advancements enable researchers to systematically test and validate model predictions, bridging the gap between theoretical optimization and experimental realization. The integration of organ-on-a-chip platforms and patient-derived organoids can also provide biologically relevant validation environments for next-generation models, enabling iterative improvements based on direct feedback from physiologically representative systems [97].

Looking ahead, the field would benefit from a greater emphasis on personalized medicine, where models incorporate patient-specific parameters to guide therapy selection and dosing. As models become more complex and computational methods advance, there is potential for real-time clinical decision support, where healthcare providers can leverage predictive simulations to adjust treatment plans dynamically. These developments, coupled with ongoing innovations in nanoparticle design and functionalization (e.g., stimuli-responsive, immunomodulatory, or multi-drug loaded nanoparticles), are set to expand the toolbox for improving drug targeting, increasing therapeutic efficacy, and minimizing off-target effects.

In summary, the future of nanoparticle-based cancer drug delivery and associated mathematical modeling is poised to become increasingly data-driven, patient-centric, and integrative. By embracing these emerging technologies and methodologies, the field can advance toward more robust, accurate, and clinically meaningful predictions—ultimately improving outcomes and reducing the burden of cancer therapy on patients.

## 5. Conclusions

Mathematical modeling plays a critical role in advancing nanoparticle-based cancer drug delivery by improving the understanding and prediction of drug behavior. However, to move beyond current limitations, researchers should prioritize a few key strategies. First, integrating comprehensive, high-quality datasets from diverse sources—including multi-omic profiles, advanced imaging modalities, and patient-derived tissues—can provide richer inputs for models. Collaborations with experimentalists can ensure ongoing feedback loops that refine parameter estimates and validate model predictions. Second, adopting multi-scale modeling frameworks that span molecular interactions, cellular uptake, and whole-organism pharmacokinetics will capture the full complexity of nanoparticle dynamics. Third, employing AI-driven simulations and machine learning tools can enable adaptive models that incorporate evolving datasets and patient-specific parameters, improving both accuracy and personalization. Finally, establishing standardized protocols and shared databases can facilitate model comparison, reproducibility, and community-driven improvements. By taking these concrete steps, the research community can enhance the predictive power and clinical relevance of mathematical models, ultimately guiding the design of more effective and personalized nanoparticle-based cancer therapies.
